# Cyclosporin A protects JEG-3 cells against oxidative stress-induced apoptosis by inhibiting the p53 and JNK/p38 signaling pathways

**DOI:** 10.1186/s12958-020-00658-0

**Published:** 2020-10-12

**Authors:** Bin He, Qi Yue Li, Yuan Yuan Wu, Jing Ling Ruan, Xiao Ming Teng, Da Jin Li, Chuan Ling Tang

**Affiliations:** 1 Department of Reproductive Medicine, Shanghai First Maternity and Infant Hospital, Tongji University School of Medicine, Shanghai, 201204 China; 2 Laboratory for Reproductive Immunology, Hospital and Institute of Obstetrics and Gynecology, Fudan University Shanghai Medical College, Shanghai, 200011 China

**Keywords:** Apoptosis, Oxidative stress, Trophoblast, p53, MAPK

## Abstract

**Background:**

Trophoblast cells are required for the establishment of pregnancy and fetal development. Apoptosis is an essential feature for trophoblast invasion. Uncontrolled trophoblast apoptosis is related to some complicate pregnancies. Oxidative stress (OS) is an important inducer of trophoblast apoptosis. Cyclosporin A (CsA) has been shown to promote the activity of trophoblast cells and reduce OS-induced oxidative injury. We investigated the role and mechanism of CsA in oxidative stress-induced trophoblast cell apoptosis.

**Methods:**

JEG-3 cells were cocultured with H_2_O_2_ and CsA. Cell viability and morphology were measured by MTT assay and DAPI staining. Cell apoptosis was tested with annexin V/PI staining. The expression of Bcl-2-associated X protein (Bax), B-cell lymphoma/leukemia-2 (Bcl-2), cleaved poly (ADP-ribose) polymerase (PARP) and pro-caspase-3 was assayed by western blotting. The protein expression and phosphorylation of p53 and mitogen-activated protein kinase (MAPK) kinases (JNK, ERK1/2 and p38) were examined by western blotting.

**Results:**

CsA increased the viability, alleviated morphological injury and reduced cell apoptosis of the H_2_O_2_-treated JEG-3 cells. CsA also attenuated the activation of p53, decreased the expression of Bax and cleavage of PARP, and increased the expression of Bcl-2 and pro-caspase-3 in the JEG-3 treated with H_2_O_2_. Furthermore, CsA reduced the activation of JNK and P38 but had no significant effect on the activation of extracellular signal-regulated kinase 1/2 (ERK1/2) in the H_2_O_2_-treated JEG-3 cells. Promoting the activation of JNK and p38 impaired the protective effect of CsA on OS-induced trophoblast apoptosis.

**Conclusions:**

These results suggested that CsA protected trophoblast cells from OS-induced apoptosis via the inhibition of the p53 and JNK/p38 signaling pathways.

## Introduction

Blastocyst implantation is mediated by the proper and strictly controlled invasion of extravillous trophoblasts [1]. The proliferation, differentiation and invasion of trophoblast cells are required for the establishment of pregnancy and fetal development. The impairment of these processes in trophoblasts may lead to pregnancy-related diseases, such as miscarriage, fetal growth restriction, and preeclampsia [2]. Successful trophoblast invasion in normal pregnancy relies on an adequate interaction between trophoblast cells and maternal epithelial, immune and endothelial cells and tissues [3]. The maternal immune system plays a key role in the processes of trophoblast invasion [4].

An important mechanism involved in maternal-fetal immune tolerance is apoptosis [5]. During healthy and abnormal placentation, apoptosis regulates the number and type of trophoblasts populating the placenta and decidua, greatly influencing maternal-fetal immune tolerance [6]. On the one hand, apoptosis is particularly important in the initial and final stages of placental development and function. The apoptotic cells are associated with stages of placental development, including the differentiation, migration and invasion of trophoblasts [7]. On the other hand, uncontrolled trophoblast cell apoptosis has a significant negative impact on placental development. Increased trophoblast apoptosis and reduced cell invasion were observed in pregnancies marked by hypertension or preeclampsia [8]. Indeed, several studies have shown that an early stage of apoptosis is activated and specifically targets cytotrophoblasts, whereas later stages of apoptosis are more likely to affect syncytiotrophoblasts and the presence of syncytial knots [9]. Syncytiotrophoblast apoptosis in preeclampsia has also been associated with larger numbers of syncytial knots and higher oxidative stress [10].

Oxidative stress is an imbalance between reactive oxygen species (ROS) generation and antioxidant defense, which is closely related to complicated pregnancies [11]. Excess production of ROS leads to a disturbance of redox potential that, in turn, causes oxidative stress and functional injury to trophoblast cells [12]. Oxidative stress can induce apoptosis via the Fas/caspase-8 and mitochondria/caspase-9 pathways. It can also modify some key apoptotic regulators, such as the B-cell lymphoma/leukemia-2 (Bcl-2), p53, c-JNK (c-Jun N-terminal kinases), and p38 MAPK (mitogen-activated protein kinase) proteins [13]. In support of this hypothesis, exaggerated apoptosis can be reproduced in trophoblasts in vitro by exposing them to hypoxia and reactive oxygen species [14]. Resveratrol inhibited the trophoblast apoptosis induced by oxidative stress in a preeclampsia rat model [15].

Cyclosporin A (CsA) is a revolutionary immunosuppressant widely used for treatments associated with organ transplantation and autoimmune disease [16]. In addition to its role in the immune system, CsA has favorable effects on maternal–fetal interface modulation [17]. Low-dose CsA can promote the proliferation and invasion of normal human first-trimester trophoblast cells [18]. Results from our previous studies showed that H_2_O_2_ induced trophoblast oxidative stress and apoptosis through the MAPK signaling pathway [19]. Low-dose CsA protects trophoblasts from H_2_O_2_-induced oxidative injury via the FAK-Src signaling pathway [20]. These results indicate that CsA can protect trophoblasts from oxidative stress-induced injury. However, whether CsA has any potential benefit on oxidative stress-induced trophoblast apoptosis is unclear. Understanding the role and molecular mechanism of CsA in oxidative stress-induced trophoblast apoptosis is very important for the clinical application of CsA to pregnancy disorders.

In this study, we demonstrated that H_2_O_2_ can induce the apoptosis of human trophoblast-like JEG-3 cells. Therefore, focusing on the apoptosis mechanism, we undertook this study to investigate whether CsA can protect trophoblasts from oxidative stress-induced apoptosis and to discern the detailed signaling pathway involved in this process.

## Materials and methods

### Cell culture

Choriocarcinoma JEG-3 (human trophoblast-like cell line) cells were obtained from the Cell Bank of the Chinese Academy of Sciences (Shanghai, China; original source: American Type Culture Collection (ATCC)). The cells were cultured in DMEM/F12 complete medium supplemented with 10% fetal bovine serum (FBS) and maintained in 5% CO_2_ at 37 °C.

### Cell viability assay

JEG-3 cells were cultured in 96-well flat-bottom microplates seeded with approximately 2 × 10^4^ cells/well. H_2_O_2_ (500 μM) was added to the cells with or without CsA (Sigma, Darmstadt, Germany) pretreatment. To induce the activation of p38 and JNK, the cells were treated with their agonists hesperetin (40 μM, MedChemExpress, NJ, USA) and anisomycin (10 μM, MedChemExpress, NJ, USA), respectively. Then, the MTT reagent (Sigma, Darmstadt, Germany) was added to each well and incubated for 4 h at 37 °C. Subsequently, the medium was removed, and 150 ml dimethyl sulfoxide was added and incubated at room temperature for 30 min. The formazan absorbance was measured at a wavelength of 490 nm on an automatic microplate reader (Bio-Rad, CA, USA).

### Cell morphology

JEG-3 cells were pretreated with CsA for 24 h and then incubated with 500 μM H_2_O_2_. To induce the activation of p38 and JNK, the cells were treated with their agonists hesperetin and anisomycin, respectively. The cells were collected and treated with 4% paraformaldehyde for 20 min. Then, the cells were washed and stained with DAPI (Sigma, Darmstadt, Germany) for 5 min. Cell morphology was observed by fluorescence microscopy (Olympus, Japan).

### Cell apoptosis assay

JEG-3 cells were pretreated with CsA for 24 h and then incubated with 500 μM H_2_O_2_. The cells were collected, and the apoptosis ratio was quantified by flow cytometry with a commercially available annexin V-FITC apoptosis detection kit (Invitrogen, CA, USA) according to the manufacturer’s guidelines. The experiments were performed in triplicate and repeated three times.

### Western blotting

JEG-3 cell lysates were prepared. Protein samples (50 μg) were separated by 10% SDS-PAGE and transferred onto nitrocellulose membranes. After blocking, the membrane was probed overnight with specific primary monoclonal rabbit anti-p-p53 (Ser15), anti-p53, anti-Bax (Bcl-2-aAssociated X protein), anti-Bcl-2, anti-pro-caspase-3, anti-cleaved PARP (Asp214), anti-p-p38 (Thr180/Tyr182), anti-p38, anti-p-ERK (Thr202/Tyr204), anti-ERK, anti-p-JNK (Thr183/Tyr185), anti-JNK (Cell Signaling Technology, MA, USA), and monoclonal mouse anti-GAPDH (Santa Cruz, TX, USA) antibodies at 4 °C and then incubated with horseradish peroxidase-conjugated secondary antibodies. Bands were visualized using an ECL detection system (Thermo Scientific, CA, USA) and quantified by densitometry.

### Statistical analysis

Independent experiments were performed at least three times. The results are presented as the means ± SEM. Statistical comparisons were performed by one-way analysis of variance followed by *Dunnett’s* test. Differences were considered statistically significant at *p* < 0.05.

## Results

### CsA attenuated H_2_O_2_-damaged JEG-3 cell viability and morphology

**Fig. 1:**
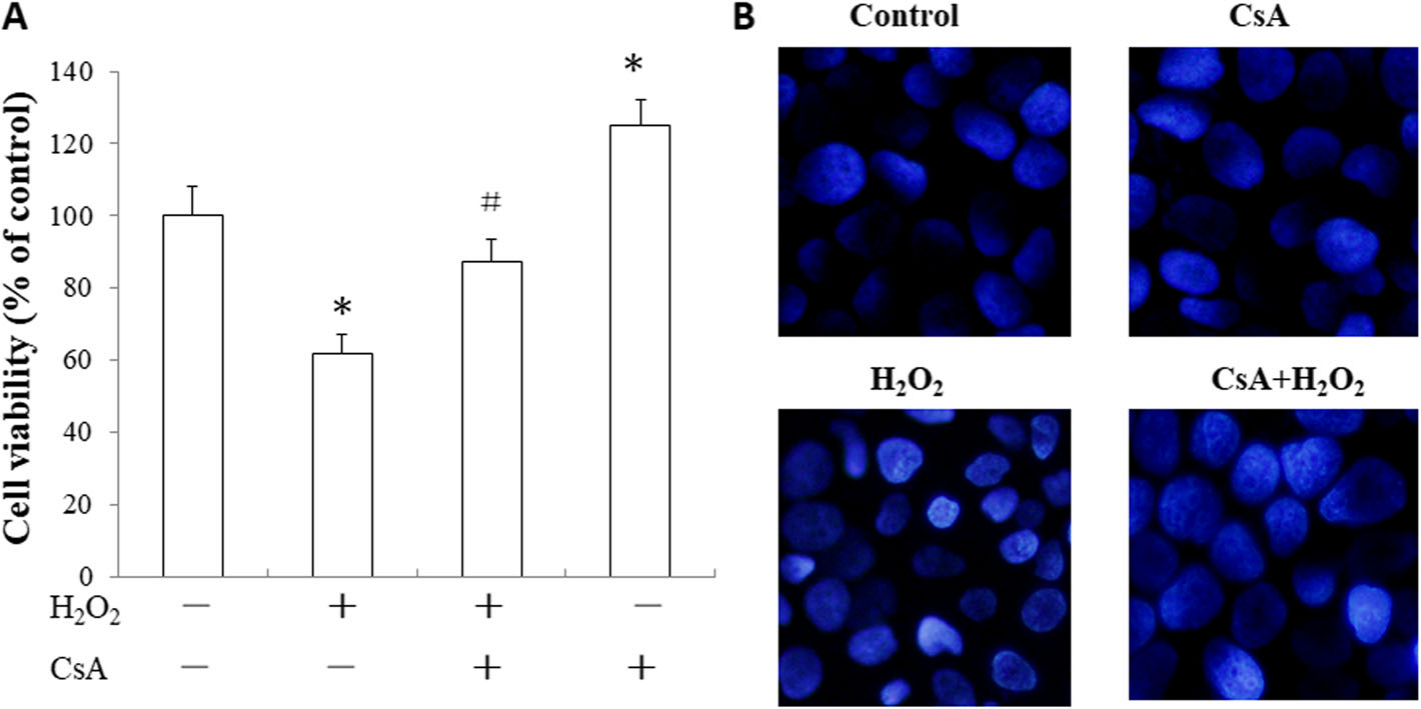
CsA protects JEG-3 cells against H_2_O_2_–induced cytotoxicity. **a** JEG-3 cells were pretreated with 1 μM CsA for 24 h followed by treatment with 500 μM H_2_O_2_ for another 24 h. Cell viability was measured by MTT assay. Error bars depict the SEM. *p < 0.05, compared to the control; ^#^ *p* < 0.05, compared to the H_2_O_2_-treated group. **b** As observed with DAPI staining with a fluorescence microscope (original magnification 200×), the H_2_O_2_-treated cells exhibited nuclear condensation and fragmentation. The data presented are representative of three independent experiments

H_2_O_2_ (500 μM) significantly suppressed cell viability. However, CsA (1 μM) pretreatment for 24 h attenuatedthe cells damage induced by H_2_O_2_, increasing their viability (*p* < 0.05, Fig. 1a). DAPI staining observed under a fluorescence microscope (original magnification 200×) revealed that the H_2_O_2_-treated cells exhibited nuclear condensation and fragmentation. In cells pretreated with CsA, the injury was clearly attenuated (Fig. 1b).

### CsA attenuated the H_2_O_2_-induced apoptosis of JEG-3 cells

**Fig. 2:**
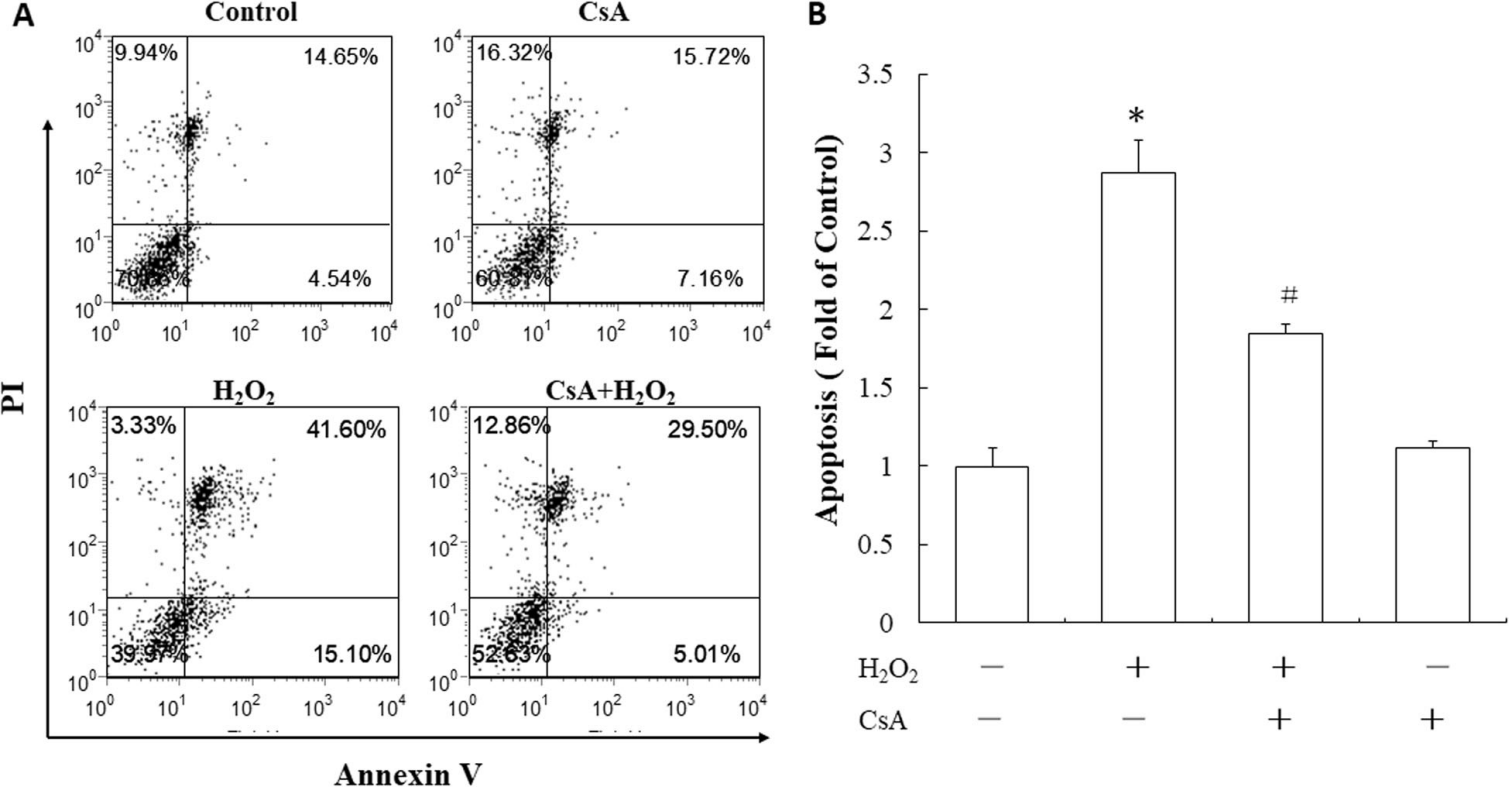
CsA attenuates H_2_O_2_-induced apoptosis in JEG-3 cells. **a** JEG-3 cells were pretreated with 1 μM CsA for 24 h followed by treatment with 500 μM H_2_O_2_ for another 24 h. Cells were collected and stained with annexin V and PI for apoptosis analysis by flow cytometry. **b** Quantitative presentation of the data (mean + SD of three samples) in percentage of apoptotic cells. The data presented are representative of three independent experiments. *p < 0.05, compared to the control; ^#^ *p* < 0.05, compared to the H_2_O_2_-treated group

To further investigate the role of CsA on H_2_O_2_-induced trophoblast apoptosis, JEG-3 cells were treated with CsA and H_2_O_2_. Cell apoptosis was assayed with annexin V/PI staining. As shown in Fig. 2. H_2_O_2_ treatment significantly increased the apoptosis rate of the JEG-3 cells. However, CsA pretreatment reduced the H_2_O_2_-induced cell apoptosis rate.

### CsA increased Bcl-2 expression and reduced Bax and p53 expression in the H_2_O_2_-treated JEG-3 cells

**Fig. 3:**
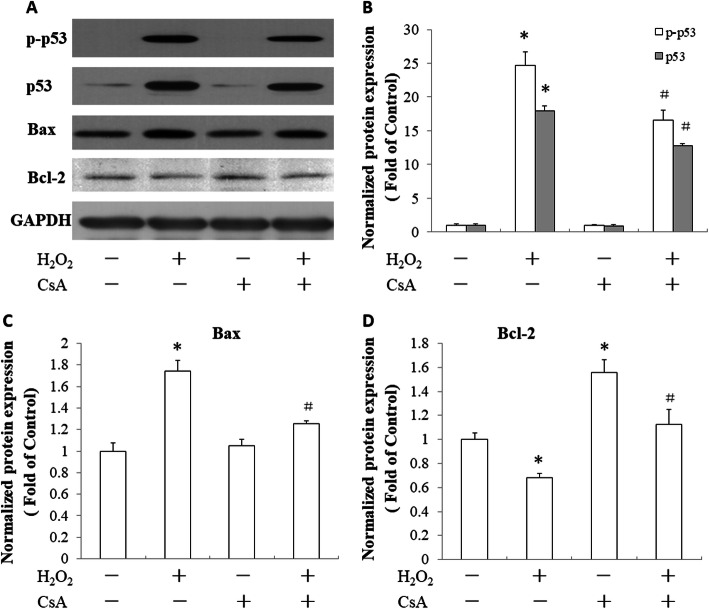
Effects of CsA on H_2_O_2_-induced expression of p-p53, p53, Bax and Bcl-2 in JEG-3 cells. **a** JEG-3 cells were pretreated with 1 μM CsA for 24 h followed by treatment with 500 μM H_2_O_2_ for another 24 h. Total cell lysates were harvested for western blot analysis of p-p53, p53, Bax and Bcl-2 expression. GAPDH was used as a loading control. **b** Quantitative analysis of p-p53 and p53 expression levels. **c** Quantitative analysis of Bax expression levels. **d** Quantitative analysis of Bcl-2 expression levels. The data presented are representative of three independent experiments. *p < 0.05, compared to the control; ^#^ *p* < 0.05, compared to the H_2_O_2_-treated group

Bax, Bcl-2 and p53 genes play important roles in modulating cell apoptosis. H_2_O_2_ treatment increased the expression of p53 and Bax and the phosphorylation of p53. In addition, it reduced the expression of Bcl-2 in the JEG-3 cells (Fig. 3). We observed the effect of CsA on the expression of these apoptosis-related genes in H_2_O_2_-treated JEG-3 cells. As shown in Fig. 3, CsA pretreatment reduced the expression of p53 (Fig. 3a, b) and BAX (Fig. 3a, c), decreased the phosphorylation of p53 (Fig. 3a, b) and increased the expression of BCL-2 (Fig. 3a, d) in the H_2_O_2_-treated JEG-3 cells.

### CsA reduced the level of cleaved PARP and increased the expression of pro-caspase-3 in the H_2_O_2_-treated JEG-3 cells

**Fig. 4:**
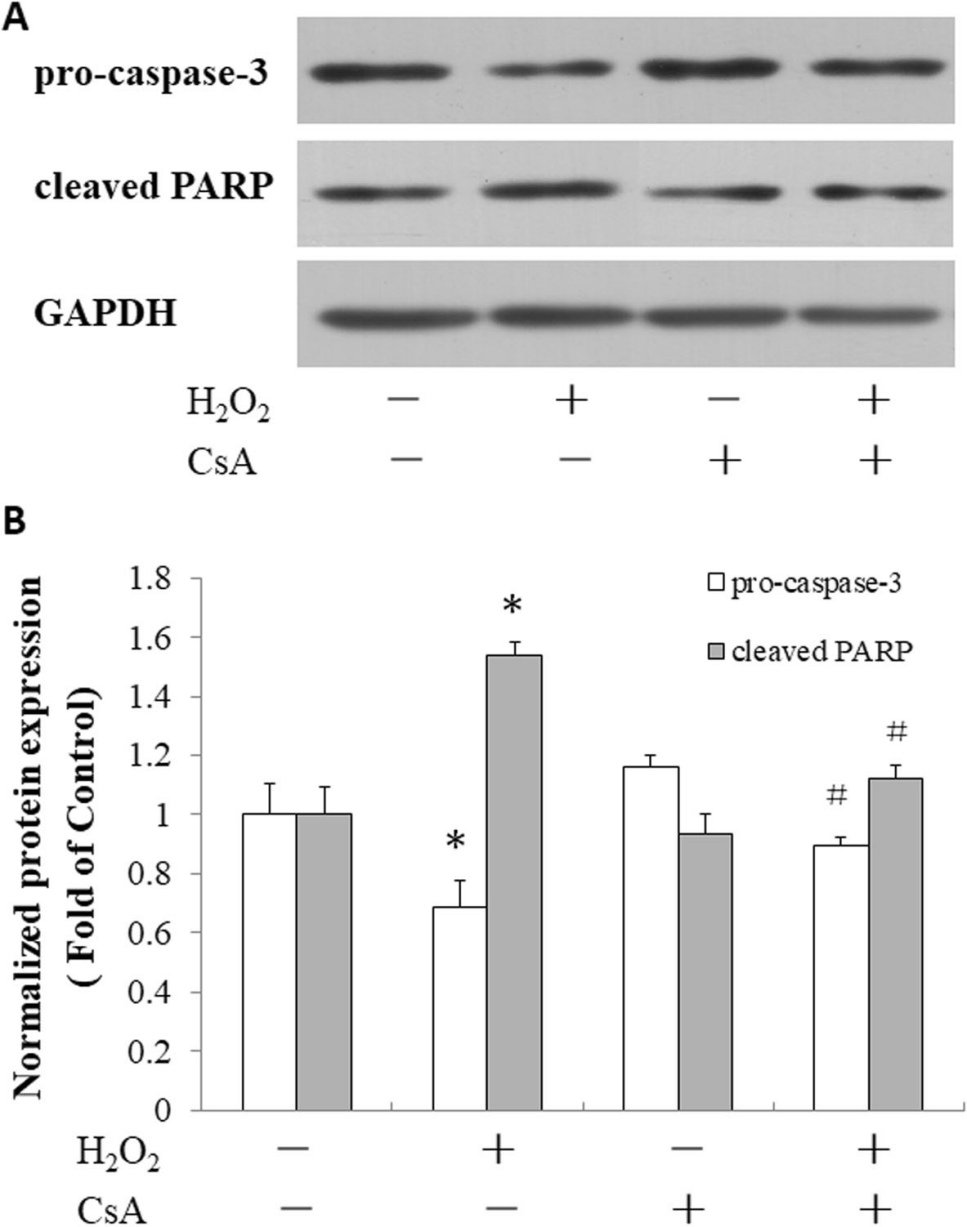
Effects of CsA on H_2_O_2_-induced expression of pro-caspase-3 and cleaved PARP in JEG-3 cells. **a** JEG-3 cells were pretreated with 1 μM CsA for 24 h followed by treatment with 500 μM H_2_O_2_ for another 24 h. Total cell lysates were harvested for the western blot analysis of pro-caspase-3 and cleaved PARP expression. GAPDH was used as a loading control. **b** Quantitative analysis of pro-caspase-3 and cleaved PARP expression levels. The data presented are representative of three independent experiments. *p < 0.05, compared to the control; ^#^ *p* < 0.05, compared to the H_2_O_2_-treated group

We further assayed the effect of CsA on the levels of pro-caspase-3 and cleaved PARP in the H_2_O_2_-treated JEG-3 cells. H_2_O_2_ treatment reduced the expression of procaspase-3 and increased the level of cleaved poly (ADP-ribose) polymerase (PARP) in the JEG-3 cells. Pretreatment with CsA reduced the level of cleaved PARP and increased the expression of pro-caspase-3 in the H_2_O_2_-treated JEG-3 cells (Fig. 4).

### CsA protected trophoblast cells from H_2_O_2_-induced apoptosis by inhibiting the p38 and JNK signaling pathways

**Fig. 5:**
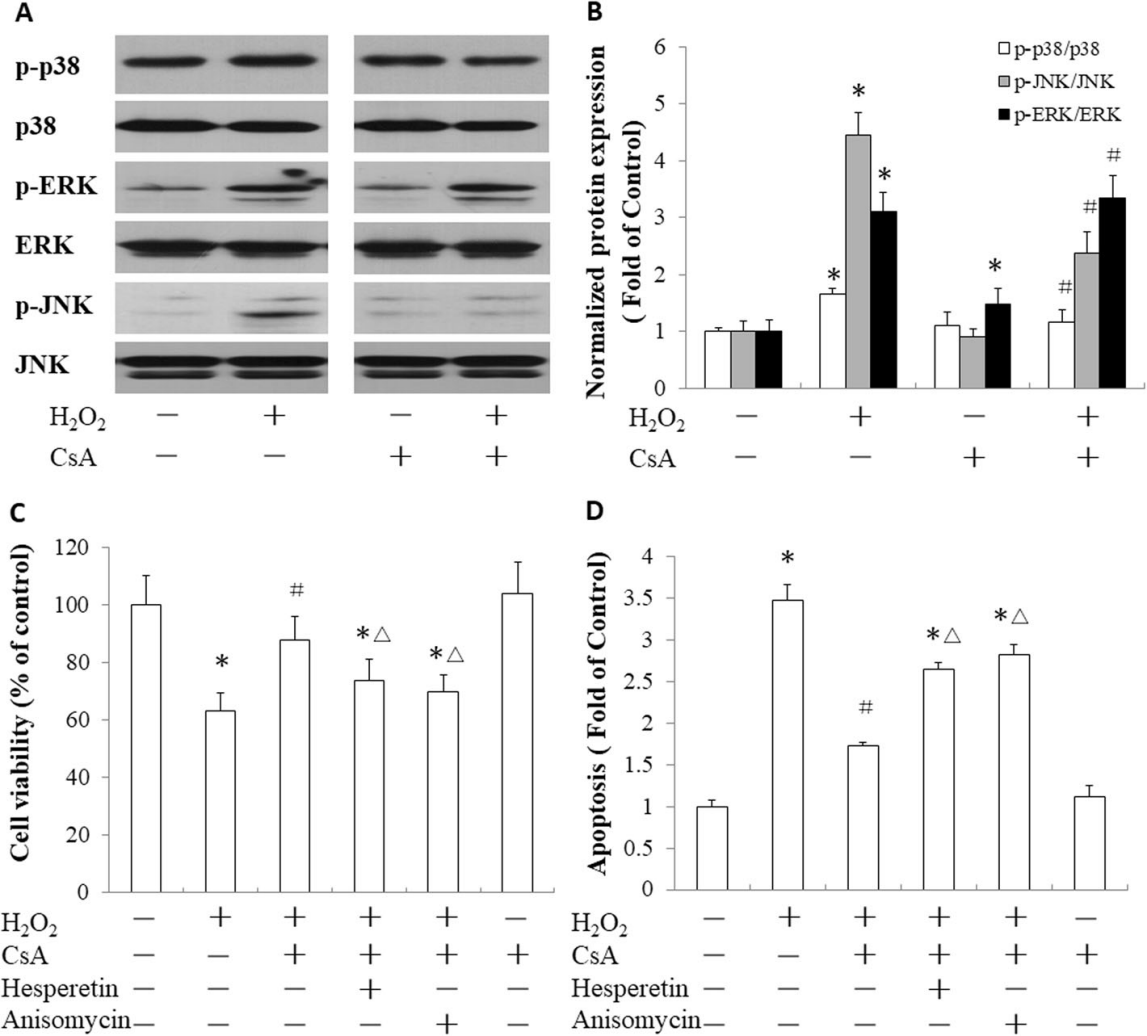
Roles of CsA in H_2_O_2_-induced activation of p38, JNK and ERK1/2 in JEG-3 cells. **a** JEG-3 cells were pretreated with 1 μM CsA in serum-free medium for 24 h and then stimulated with 500 μM CsA for 15 min. The phosphorylation of p38, JNK and ERK1/2 was evaluated by western blot analysis. **b** Quantitative analysis of the phosphorylation levels of p38, JNK and ERK1/2. **c** The viability of the JEG-3 cells after treatment with hesperetin or anisomycin. **d** Apoptosis of the JEG-3 cells after treatment with hesperetin or anisomycin. The data presented are representative of three independent experiments. *p < 0.05 compared to the control; ^#^ *p* < 0.05 compared to the H_2_O_2_-treated group; ^△^ *p* < 0.05 compared to the group treated with CsA and H_2_O_2_

To further investigate the mechanism of CsA protection against H_2_O_2_-induced trophoblast apoptosis, we analyzed the phosphorylation of p38, JNK and ERK1/2 in JEG-3 cells. H_2_O_2_ treatment resulted in the phosphorylation of p38, JNK and ERK1/2 in the JEG-3 cells. CsA pretreatment reduced the activation of p38 and JNK in the H_2_O_2_-treated JEG-3 cells, but it had no effect on the activation of ERK1/2 in the cells treated with H_2_O_2_ (Fig. 5a, b)_._ To confirm the role of the p38 and JNK signaling pathways in the protection of trophoblast cells from H_2_O_2_-induced apoptosis by CsA, JEG-3 cells were treated with hesperetin or anisomycin to promote the activation of p38 or JNK, respectively. These activators blocked the activity of CsA, decreased cell viability (Fig. 5c) and increased the cell apoptosis rate (Fig. 5d). These results indicated that CsA protected JEG-3 cells from H_2_O_2_-induced apoptosis via the inhibition of the p38 and JNK signaling pathways.

## Discussion

Cyclosporin A (CsA) is a lipophilic cyclic peptide of 11 amino acids and was first discovered in the fungus in 1970 [21]. High-dose CsA is a very potent and relatively selective inhibitor of T lymphocyte activation and is used as an immunosuppressive drug and anti-rejection drug in solid organ transplantation [16]. Long-term treatment with CsA is associated with many side effects, including nephrotoxicity, neurotoxicity, and cardiovascular toxicity. Therefore, the current tendency is to decrease its dose. The literature has reported data revealing a paradoxical effect of low dosage of cyclosporin-A (≤3 mg/kg), which appears to have immunomodulatory properties [22]. In addition to its role in the immune system, low-dose CsA has been reported to improve pregnancy outcomes. According to the US Food and Drug Administration, CsA is classified as a class C drug for pregnancy. An appropriate dose of CsA (from 80 ng/ml to 150 ng/ml) produces good curative effects and, in pregnancy, is used to treat immunity-related recurrent spontaneous abortion [23]. A low-dose CsA (150 mg/day) treatment for 6 months improves pregnancy outcomes in women with recurrent pregnancy loss and an elevated Th1/Th2 ratio [24]. Low-dose CsA (100 mg/day) treatment for 30 days increases the live birth rate for women with unexplained recurrent abortion, and it produces no obvious side effects or adverse consequences [25]. Low-dose CsA promotes the growth and invasiveness of human first-trimester trophoblast cells in vitro via MAPK3/MAPK1-mediated AP1 and Ca^2+^/calcineurin/NFAT signaling pathways; however, high-dose CsA shows the opposite effect [18]. According to these results, low-dose CsA was used for current study.

During pregnancy, the development of the placenta is interrelated with the oxygen concentration. Pregnancy disorders are often associated with excessive oxidative stress, which induces trophoblast apoptosis and impairs trophoblast differentiation, migration and invasion [13, 26]. Trophoblast apoptosis is a major consequence of oxidative stress and causes some complicated pregnancies [6]. CsA has been demonstrated to have favorable effects on trophoblast activity and maternal–fetal interface modulation. Our previous study showed that low-dose CsA protects trophoblasts from H_2_O_2_-induced oxidative injury via the FAK-Src signaling pathway [20]. In the current study, we also found that low-dose CsA pretreatment attenuated H_2_O_2_ damage to maintain the viability and morphology of the JEG-3 cells. However, the mechanism of action of CsA on oxidative stress-induced trophoblast apoptosis is not clear.

Apoptosis is a physiological phenomenon in trophoblast turnover. The apoptosis frequency in placental villi is lowest in the first trimester, increasing in the third trimester, and markedly accelerated after 40 weeks gestation [27, 28]. However, increased levels of apoptotic markers in trophoblasts have been identified in placental pathologies [6, 28]. Oxidative stress (OS) is an important inducer of trophoblast cell apoptosis [26]. H_2_O_2_ is a stable member of the ROS family, which plays important roles in oxidative stress-mediated diseases. In our previous study, H_2_O_2_ treatment induced oxidative damage in trophoblast cells, resulting in reduced cell viability and increased ROS generation [19]. In this study, H_2_O_2_ treatment induced trophoblast cell apoptosis, as determined with annexin V/PI staining. Furthermore, CsA pretreatment reduced the H_2_O_2_-induced apoptosis rate of JEG-3 trophoblast cells. This result indicated that CsA protected trophoblast cells from oxidative stress-induced apoptosis.

OS can induce apoptosis via extrinsic and intrinsic signals. The former is mediated by the FAS receptor on the cell surface upon binding of its FAS ligand, which results in the activation of the caspase-8 signal; the latter is activated during intracellular stress, such as ischemia and DNA damage, and is induced from signals transmitted by mitochondria-mediated caspase-9 pathways [29]. The tumor suppressor p53 is an important sensor of OS, and the induction of oxidative stress is generally accompanied by the activation of p53. Additionally, p53 activation functions as a pro-oxidant factor that may further trigger oxidative stress [30]. During intracellular stress, p53 is also activated and stimulates cascades of proapoptotic factors mainly targeting the mitochondria [31]. Regarding the apoptotic function of the intrinsic pathway, p53 also modulates both prosurvival and proapoptotic Bcl-2 family members. Following DNA damage, the tumor suppressor p53 is posttranslationally activated, and activated p53 transcriptionally upregulates PUMA and NOXA, important proteins of the Bcl-2 family, and then induces the apoptosis mechanism [32, 33]. H_2_O_2_ treatment induced trophoblast cell apoptosis by increasing its expression and phosphorylation, and CsA pretreatment reduced H_2_O_2_-induced p53 expression and activation, which demonstrated that CsA prevented OS-induced trophoblasts by inhibiting the expression and activation of p53 transcriptional factors.

Oxidative stress is one trigger of the intrinsic mitochondrial pathway. Subsequently, it promotes the activation of the proapoptotic factors Bax and Bak, which neutralizes the anti-apoptotic Bcl-2 and Bcl-xL proteins, leading to the disruption of mitochondrial membrane permeability (MMP) [13, 34]. The intrinsic pathway is regulated by Bcl-2 family proteins. The Bcl-2 proteins are classified into three subgroups (BH1, BH2 and BH3): one group with anti-apoptotic function includes Bcl-2 and Bcl-xL, and. The other two groups with proapoptotic function include Bax, Bak, Bid, the PUMA proteins [32, 34].. Balanced protein interactions between Bcl-2 family members are required to determine cell survival or apoptosis. The activation of Bax/Bak triggers the alteration of mitochondrial membrane permeability. The proapoptotic BH3-only proteins bind to anti-apoptotic Bcl-2 family members, thereby enabling Bax/Bak to elicit MMP and activate the caspase cascade [35, 36]. In the JEG-3 trophoblast cells, H_2_O_2_ induced higher expression of Bax and reduced the expression of Bcl-2 protein. However, CsA treatment promoted the expression of the Bcl-2 protein and decreased the expression of the Bax protein. These results indicated that H_2_O_2_ triggered the intrinsic apoptosis pathway. CsA protected trophoblast cells from OS-induced apoptosis by modulating the balance of the Bax/Bcl-2 proteins.

Apoptosis is primarily executed by caspase proteases [37]. Caspases are central factors in the mechanism of apoptosis. Some caspases are initiators of apoptotic pathways, such as caspase-2, − 8, − 9 and − 10, and some are executors of apoptosis, such as caspase-3, − 6 and − 7 [38]. Once activated, the initiator caspases cleave the executor caspases, which then perform critical cleavage of specific cellular substrates, resulting in apoptotic cell death [39]. Subsequent activation of the proapoptotic activity of Bax/Bak induces the expression of apoptogenic factors, such as cytochromec, triggering the formation of apoptosomes and autoactivation of pro-caspase-9. This process, in turn, activates downstream executors, caspase-3, − 6 and − 7 that cleave cellular substrates, such as PARP, leading to apoptotic cell death [40]. The results from this study showed that H_2_O_2_ increased the level of cleaved PARP and reduced the level of pro-caspase-3. CsA pretreatment led to the opposite result in H_2_O_2_-treated trophoblasts, which indicated that CsA reduced the activation of the caspase cascades.

The MAPK (JNK, ERK, and p38) signaling pathway involves a family of serine/threonine protein kinases that regulate cell proliferation, cell survival, and apoptosis. They play important roles in OS-induced apoptosis [41]. ERK is primarily activated by the exposure of cells to cytokines and growth factors. In contrast, p38 and JNK respond primarily to extracellular and intracellular stress [42]. JNK activates the proapoptotic Bcl-2 protein family members Bim and Bax, eliciting MMP and OS-induced apoptosis [43]. The p38 protein is activated by ROS and can induce apoptosis following the stimulation of TNF-α, the recruitment of caspase-8 and caspase-3, and the activation of the downstream effector protein PARP [44]. To understand the activity of the MAPK signaling pathway in CsA protection against OS-induced trophoblast apoptosis, the phosphorylation of JNK, ERK, and p38 was detected by western blotting. JNK, ERK, and p38 were activated to high levels upon H_2_O_2_ treatment. CsA reduced the phosphorylation of JNK and p38, but it had no effect on the activation of ERK in the JEG-3 cells treated with H_2_O_2_. Promoting the activation of p38 and JNK with hesperetin or anisomycin impaired the protective activity of CsA on OS-induced trophoblast apoptosis. These results indicated that CsA reduced OS-induced trophoblast apoptosis by inhibiting the JNK/P38 signaling pathway.

## Conclusions

In summary, apoptosis is an essential feature for trophoblast invasion and potentially plays a role in maternal immune tolerance. Uncontrolled trophoblast apoptosis is related to some complications in pregnancy. Oxidative stress is a major factor that induces trophoblast apoptosis. In JEG-3 cells with H_2_O_2_-induced oxidative stress, CsA treatment reduced OS-induced trophoblast cell viability and apoptosis; reduced the activation of p53, JNK and p38; decreased the expression of BAX and cleaved PARP; and increased the expression of Bcl-2 and pro-caspase-3. These results suggested that CsA protected trophoblast cells from OS-induced apoptosis via the inhibition of the p53 and JNK/p38 signaling pathways.

## Data Availability

All data generated or analyzed in this study are included in this published article.

## Abbreviations

OS: Oxidative stress
CsA: Cyclosporin A
H_2_O_2_: Hydrogen peroxide
Bax: Bcl-2-associated X protein
Bcl-2: B cell lymphoma/leukemia-2
PARP: Poly (ADP-ribose) polymerase
MAPK: Mitogen-activated protein kinase
JNK: C-Jun N-terminal kinase
ERK1/2: Extracellular signal regulated kinase 1 and 2
ROS: Reactive oxygen species
GAPDH: Glyceraldehyde-3-phosphate dehydrogenase
FAK: Focal adhesion kinase
Src: Src kinase
PUMA: p53 upregulated modulator of apoptosis
NOXA: NOXA protein
MMP: Mitochondrial membrane permeability
